# Measuring Primary Health Care Clinicians’ Skills for Depression Management

**DOI:** 10.3389/fpsyt.2019.00570

**Published:** 2019-08-14

**Authors:** Pablo Martínez, Graciela Rojas, Vania Martínez, Rigoberto Marín, Juan P. Cornejo, Víctor Gómez

**Affiliations:** ^1^Departamento de Psiquiatría y Salud Mental, Hospital Clínico Universidad de Chile, Santiago, Chile; ^2^Millennium Nucleus to Improve the Mental Health of Adolescents and Youths (Imhay), Santiago, Chile; ^3^Millennium Institute for Depression and Personality Research (MIDAP), Santiago, Chile; ^4^Escuela de Psicología, Facultad de Humanidades, Universidad de Santiago de Chile, Santiago, Chile; ^5^Centro de Medicina Reproductiva y Desarrollo Integral del Adolescente (CEMERA), Facultad de Medicina, Universidad de Chile, Santiago, Chile; ^6^Departamento de Educación en Ciencias de la Salud, Facultad de Medicina, Universidad de Chile, Santiago, Chile; ^7^Hospital Clínico Universidad de Chile, Santiago, Chile

**Keywords:** primary health care, depression, medical education, clinical competence, standardized patients

## Abstract

**Introduction:** Primary health care clinicians play an important role in the management of depression. Thus, it is very important to have a valid and reliable assessment of the competences needed to manage depression in primary health care, with the use of clinical simulation providing such an opportunity.

**Objective:** The present study describes the assessment of primary health care clinicians’ depression-related skills through a series of objective structured clinical examination stations.

**Material and Methods:** Clinicians from multi-professional teams for the management of depression at two primary health care clinics in Santiago, Chile, went through seven objective structured clinical examination stations, lasting 10 to 20 min each, to assess their depression-related skills. The clinical and communicative skills measured were in accordance with clinical guidelines. Standardized patients portrayed cases usually encountered in clinical practice, while expert raters evaluated clinicians’ performance with standardized checklists.

**Results:** Psychosocial clinicians performed better than biomedical clinicians in the assessed skills. The most notable results were as follows: a high level of accomplishment in the relationship with patient, medical anamnesis, health checkup, and lab test requests; heterogeneous performance in patient management according to screening results, feedback to the patient, and registration in clinical records; and major deficiencies in the differential diagnosis of bipolar disorder.

**Discussion:** The objective structured clinical examinations administered provided an opportunity to perform an in-depth examination of the depression-related skills of primary health care clinicians, where flaws in the screening and diagnosis procedures used by biomedical clinicians were detected. Given the significant involvement of these types of clinicians in depression management, undergraduate-level and continuing health education opportunities are needed.

## Introduction

Nearly 20% of adults attending urban primary health care (PHC) clinics have depression (1), one of the main public health problems globally (2). Depression has been linked to an increased risk of chronic diseases (3–5), decreased health-related quality of life (6, 7), impaired social role performance (8, 9), and excess mortality (10). Although effective depression management strategies exist (11, 12), the research-to-practice gap in the provision of mental health services has prevented a vast majority of depressed people from receiving the timely evidence-based, high quality care they need (13), leading to a higher risk of recurrence and worse outcomes (14, 15). Moreover, a lack of mental health human resources is a major contributor to the underuse of best available evidence (16).

A promising solution to these informational and financial barriers in PHC settings is to reallocate clinical responsibilities to non-specialized health care personnel with due training and supervision (17). Thus, a team-driven approach to training would be necessary to increase working capacity through collaborative care (17), a basic component for the development of effective integrated behavioral health care (18). Accordingly, in one of the regions hit hardest by depressive disorders worldwide (2), the 2013 World Health Organization Report on Mental Health Systems in Latin America and the Caribbean demonstrated the urgent need to increase the availability of training/educational opportunities for undergraduate health care students and PHC clinicians to learn about mental health subjects (19).

These mental health training programs require valid and reliable assessments of the competences of trainees—instruments that can inform future learning objectives, also known as formative assessments (20). Regarding clinical simulation [i.e., the incorporation of standardized patients into objective structured clinical examination (OSCE) stations focused on specific tasks], it has proven to be one of the most reliable, consistent, and realistic ways to measure the clinical and communication skills of health care students and clinicians (20). However, no OSCEs for assessing PHC clinicians’ management of depression have been systematized in Latin America and the Caribbean region.

In the context of a randomized clinical trial (RCT) that implemented a comprehensive training and supervision program to enhance depression management in PHC in a Southern Latin American country, the present study describes the assessment of PHC clinicians’ depression-related skills through a series of OSCE stations.

## Material and Methods

### Study Design

A descriptive secondary analysis of the intervention arm’s training component of the cluster RCT “Comprehensive Technology-Assisted Training and Supervision Program to Enhance Depression Management in Primary Care” (21) was carried out at two PHC clinics in Santiago, Chile.

The participants were clinicians who were working in multi-professional teams for the management of depression within the selected PHC clinics and gave written informed consent before entering the study. The Ethics Committee of the Faculty of Medicine, Universidad de Chile, granted approval for the study under record number 103-2012.

### Objective Structured Clinical Examinations (OSCEs)

Between April and October 2014, the PHC clinicians participating in the intervention arm were enrolled in a training program on depression management that lasted 24 teaching hours. Prior to being trained, the PHC clinicians’ depression-related skills were assessed through a series of OSCE stations.

**Figure 1:**
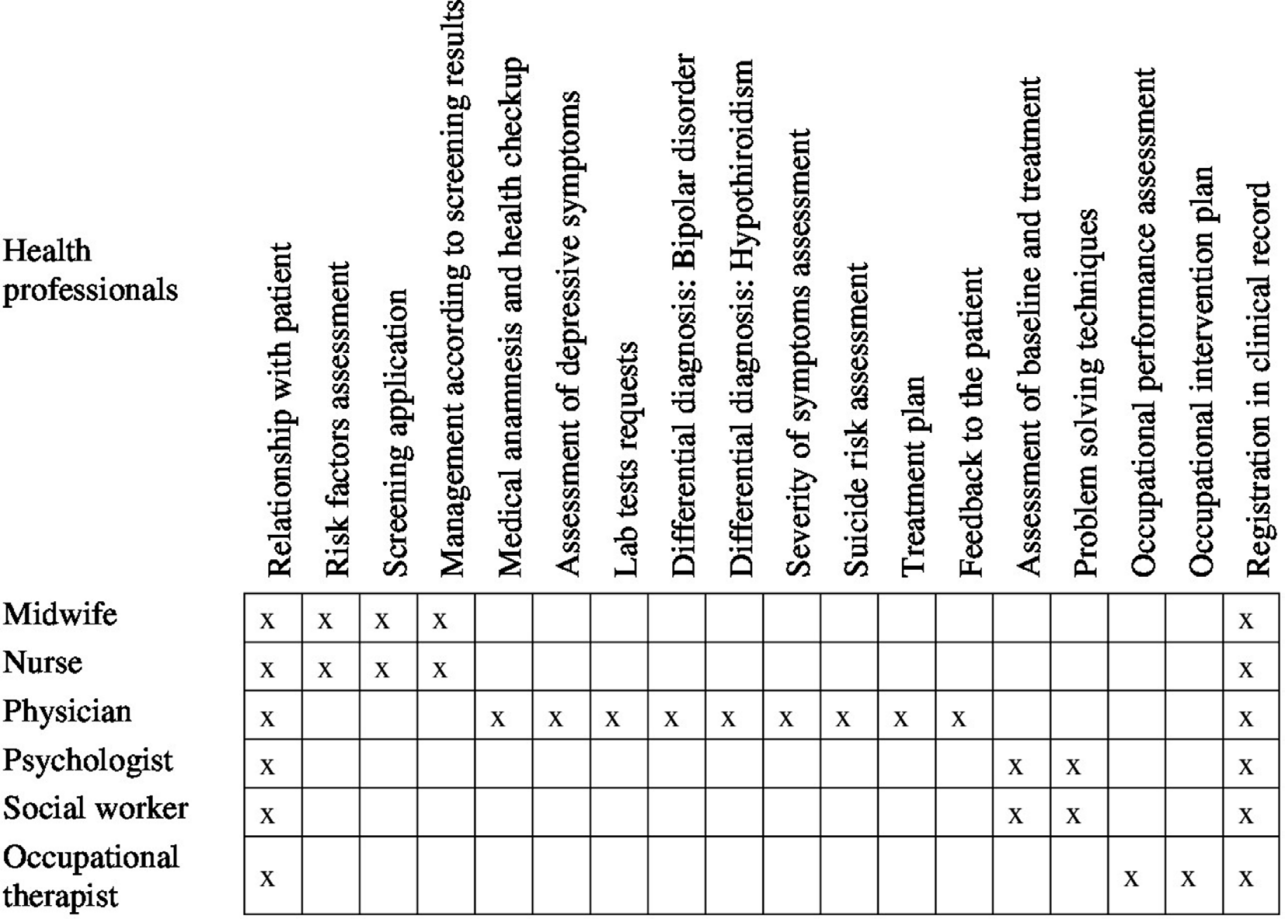
Clinical and communicative skills assessed in the PHC clinicians.

Seven OSCE stations were designed by the authors through an iterative process of discussion. Based on the National Depression Program in Chile (22), which defines the coordination among PHC clinicians and introduces a stepped-care model for depression, the clinical and communicative skills needed for the management of this condition in PHC were defined and specified for each of type of clinician (**Figure 1**). For research purposes, these skills were divided into groups of basic and observable behaviors that were used to build a checklist. This made it possible to conduct a systematic and objective assessment in which expert raters had to indicate the “presence” or “absence” of each attribute in the evaluated clinicians.

Complementarily, the scripts for each clinical scenario—i.e., OSCE station—were developed, taking into account the skills and behaviors defined above and ensuring that conditions resembled the usual clinical practice (e.g., typical profiles of depressive patients and average consultation times). Thus, specific OSCE stations were designed for each type of clinician (**Table 1**). To this end, the content of each clinical scenario was carefully reviewed by the authors and by experienced PHC clinicians; all modifications were carefully discussed by the whole research team.

**Table 1:** Brief description of OSCE stations.

Station	Task
1	Midwife must perform a screening for depression on a 19-year-old mother in the postpartum period with suspected depression, who attends her well-child check-up. Duration: 10 min
2	Nurse must perform a screening for depression on a 70-year-old patient with suspected depression who attends a routine geriatric check-up. Duration: 10 min
3	Physician must follow steps to detect and diagnose a moderate depressive episode on a 21-year-old woman who comes to a spontaneous consultation. Duration: 10 min
4	Physician must follow steps to detect and diagnose a moderate depressive episode and subclinical hypothyroidism on a 35-year-old man who is referred by another member of the PHC team. Duration: 10 min
5	Physician must create a treatment plan for a 27-year-old mother in the postpartum period who consults spontaneously and presents with a moderate depressive episode with low suicide risk. Duration: 15 min
6	The psychologist/social worker must apply problem solving techniques to help a 34-year-old woman, victim of domestic abuse, who is receiving pharmacological treatment for a depressive episode. Duration: 20 min
7	Occupational therapist must perform an evaluation of occupational performance on a 35-year-old man with a moderate depressive episode, referred by a PHC physician. Duration: 15 min

The clinical cases presented in each OSCE station were portrayed by standardized patients, defined by Barrows (23) as “[persons] carefully coached to simulate an actual patient so accurately that the simulation cannot be detected by a skilled clinician (…) [they present] the gestalt of the patient being simulated; not just the history, but the body language, the physical findings, and the emotional and personality characteristics as well.” The authors reviewed the scripts for each clinical scenario together with the standardized patients, supervising their training and giving them pointers on their nonverbal communication.

The clinical simulations were performed in the Clinical Skills Center of the Faculty of Medicine, Universidad de Chile. Three OSCE stations were implemented simultaneously, devoting a total of three teaching hours to each selected PHC clinic (i.e., PHC team). In each OSCE station, a PHC clinician entered a bay (unidirectional mirror room) where they were informed of the clinical scenario and the expected objectives through instructions placed on their desk, with the stated premise that they should behave as they usually did in clinical practice. In parallel, standardized patients moved from the actors’ room to the waiting room and waited for the coordinator’s signal to enter the bay, effectively beginning the OSCE station, which lasted between 10 and 20 min depending on the skills being assessed. The interaction was observed through the mirror by an expert rater who had a checklist for the assessment of clinical and communicative skills. Finally, the interaction between the PHC clinician and the standardized patient was interrupted by the expert rater when the time allotted to the station was over; at this point, the standardized patient left for the actors’ room and the expert rater gave feedback to the PHC clinician in the bay for 5 min.

The expert raters who evaluated the participants were experienced researchers and/or academics in the field of depression and medical education who also held degrees in topics such as mental health care in the public sector, social work, and education in the health care sciences field. The raters had no prior relationship with the participants. Lastly, to ensure the quality of their reports, the raters were informed and trained to become familiar with the OSCE stations, the procedures involved in the study, and the way in which they should assess the skills evaluated by using a checklist, which made it possible to generate results in a standardized, systematic, and objective manner.

### Results Assessment

The PHC clinicians’ performance was evaluated according to the “presence” or “absence” of a series of basic behaviors, grouped as clinical and communicative skills and specified for each OSCE station (**Figure 1**). A median of 22 basic behaviors (minimum = 15; maximum = 30) and a median of 5 clinical and communicative skills (minimum = 4; maximum = 8) were assessed per clinical scenario. The raters had a specific checklist for each OSCE station, in which the expected basic behaviors to be observed were laid out and defined. One point was assigned if the behavior was present and 0 if it was absent.

### Statistical Analysis

The PHC clinicians’ checklists were consolidated and grouped by OSCE station. Afterwards, the percentage of assessed “present” behaviors and skills was calculated for the whole of the participants in each OSCE stations. According to the percentage obtained, the following qualification rule was applied: a) “Absent”: 0–39%; b) “Less than acceptable”: 40–59%; c) “Acceptable”: 60–74%; d) “More than acceptable”: 75–100%. Descriptive analyses were assisted by SPSS 12.0.

## Results

### Sample Characteristics

The sample was composed of 41 PHC clinicians (34 women and 7 men). In **Table 2**, the distribution of the type of PHC clinicians per participating clinic is specified.

**Table 2:** Type of PHC clinicians by participating clinic.

Health professionals	PHC clinic 1	PHC clinic 2	Total
Midwives	4 (25.0%)	3 (12.0%)	7
Nurses	1 (6.2%)	3 (12.0%)	4
Physicians	5 (31.2%)	9 (36.0%)	14
Psychologists	3 (18.8%)	5 (20.0%)	8
Social workers	3 (18.8%)	3 (12.0%)	6
Occupational therapists	–	2 (8.0%)	2
Total	16	25	41

### Observed Clinical and Communicative Skills

The average number of competences assessed as “present” by station ranged from 85.7% for occupational therapists in Station 7 (“Diagnosis of occupational performance in a depressed man”) to 57.6% for nurses in Station 2 (“Screening for depression in an elderly patient”) (**Table 3**). It should be noted that occupational therapists in Station 7 and psychologists/social workers in Station 6 (“Problem-solving techniques to help a woman to cope with depression”), with 80.5% of skills being assessed as present on average, were classed as “More than acceptable,” while nurses in Station 2 and physicians—with an average of 59.2% of skills being assessed as present in Station 3 (“Comprehensive diagnosis of depression in a depressed woman”)—were ranked as “Less than acceptable.”

**Table 3:** Skills assessed as present in PHC clinicians by OSCE station.

OSCE stations	Health professionals	% (SD)
1	Midwives	72.1 (29.4)
2	Nurses	57.6 (37.3)
3	Physicians	59.2 (28.2)
4	Physicians	62.1 (31.8)
5	Physicians	77.6 (14.3)
6	Psychologists/Social workers	80.5 (16.1)
7	Occupational therapists	85.7 (23.1)

**Figure 2:**
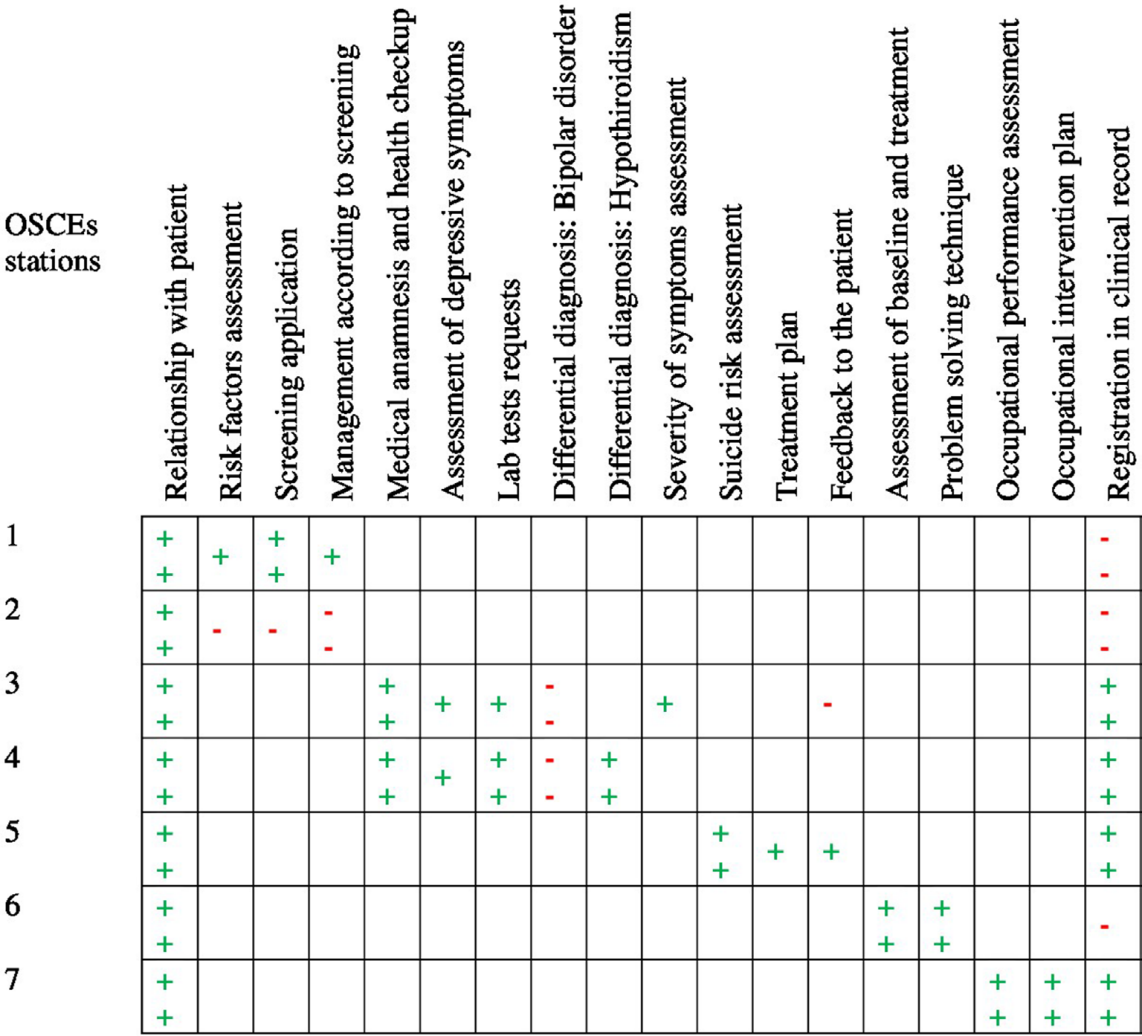
Performance in each of the skills assessed, according to OSCE stations. Symbols: ,“Absent”; , “Less than acceptable”; , “Acceptable”; and , “More than acceptable.”

In **Figure 2**, the average performance observed in each of the assessed skills is summarized by OSCE station. In Station 1 (“Screening for depression in a postpartum woman”), midwives performed at a “More than acceptable” level in the following skills: “Relationship with patient” (97.2%) and “Screening application” (76.7%), while “Registration in clinical record” (16.7%) was qualified as “Absent.” On the other hand, nurses’ performance was assessed as “More than acceptable” in the “Relationship with patient” skill (94.4%) and “Absent” for the “Management according to screening results” (33.3%) and “Registration in clinical records” (0.0%) skills, in the context of Station 2 (“Screening for depression in an elderly patient”). In addition, physicians assessed in Station 3 (“Comprehensive diagnosis of depression in a depressed woman”), were classed as “More than acceptable” in the “Registration in clinical record” (85.7%), “Medical anamnesis and health checkup” (85.7%), and “Relationship with patient” skills (78.3%); however, they received an “Absent” qualification in the “Differential diagnosis: Bipolar disorder” skill (26.6%). Also, in Station 4 (“Diagnosis of a moderate depressive disorder and subclinical hypothyroidism in a depressed man”), physicians performed at a “More than acceptable” level in the “Medical anamnesis and health checkup” (88.5%), “Lab tests requests” (85.7%), “Relationship with patient” (80.2%), “Registration in clinical record” (78.6%), and “Differential diagnosis: Hypothyroidism” (76.2%) skills, while “Differential diagnosis: Bipolar disorder” (19.4%) was found to be “Absent.” Likewise, the physicians’ performance was assessed as “More than acceptable” in the following skills: “Registration in clinical record” (92.9%), “Relationship with patient” (81.5%), and “Suicide risk assessment” (78.6%); however, they earned “Acceptable” ratings—the lowest that they obtained—in the “Feedback to the patient” (74.7%) and “Treatment plan” (71.9%) skills, in the context of Station 5 (“Treatment plan for a woman with postpartum depression with low suicide risk”).

Psychologists and social workers who were evaluated in Station 6 (“Problem-solving techniques to help a woman to cope with depression”) obtained “More than acceptable” ratings in the “Assessment of baseline and treatment” (92.9%), “Relationship with patient” (84.5%), and “Problem solving technique” (79.6%) skills, with the exception of the “Registration in clinical record” (50.0%) skill, where they performed at a “Less than acceptable” level. Lastly, in Station 7 (“Diagnosis of occupational performance in a depressed man”), occupational therapists obtained “More than acceptable” ratings in all the assessed skills: “Relationship with patient” (100.0%), “Registration in clinical record” (100.0%), “Occupational performance assessment” (83.3%), and “Occupational intervention plan” (75.0%).

## Discussion

### Main Results

After administering a series of objective structured clinical examinations (OSCEs), it was observed that PHC clinicians in Santiago, Chile, demonstrated an acceptable level of clinical and communicative skills related to the management of depression. Overall, psychosocial clinicians (psychologists, social workers, and occupational therapists) performed better than biomedical clinicians (physicians, midwives, and nurses) in the assessed skills, as the latter showed flaws linked to depression screening and diagnosis.

The most notable results were the following: a high level of accomplishment in relationship with the patient, medical anamnesis, health checkup, and lab tests requests; a heterogeneous performance in patient management according to the screening results, feedback to the patient, and registration in clinical records; and major deficiencies in the differential diagnosis of bipolar disorder and the diagnosis of depression in the presence of medical comorbidity.

### Strengths and Weaknesses of the Study

Skills for the management of depression in PHC clinicians were assessed through OSCEs, a method highlighted for its reliability and consistency in comparison to other ways of measuring clinical skills, such as clinical cases and/or questionnaires. The multi-station, against-the-clock scheme of OSCEs simulates the usual conditions of a PHC clinic, encouraging clinicians to focus on brief and precise diagnostic interviews and/or interventions. Given the problems brought up by the *in vivo* evaluation of clinical and communicative skills, OSCEs are the best choice (24).

However, this study has some important limitations. For instance, the small sample size affects the possibility of generalization. In this regard, it should be noted that this is a method for assessing clinical skills that requires significant financial and logistical resources and that the clinics that participated were representative of PHC in Chile’s capital, in administrative and professional terms. Also, future versions of this OSCE should attempt to include pre-test assessments of inter-rater agreement and—according to the informal commentary of an external collaborator—a more diligent review of the objectives declared in each station, the message delivered to the PHC clinicians, and the contents of the assessment checklist.

In addition, variations in clinical and communicative skills found between biomedical and psychosocial clinicians working in PHC may be artificial, since the participating health care professionals were subjected to different evaluation processes (i.e., multiple OSCE stations that required specific clinical skills). However, the reason for these differentiated procedures is that, in Chile, depression management in PHC is performed, by definition, by a multidisciplinary team whose members possess a distinctive set of clinical skills. Future studies, apart from increasing the number of participating clinicians, must conduct comparisons among those belonging to the same category, adjusting their results by covariates of interest.

### Comparison With Prior Work

In Latin America, there are no published studies on the use of OSCEs for assessing and/or training skills for the management of depression in PHC. Previous experiences in the region have used theoretical and attitudinal tests as well as clinical case vignettes for the assessment of these skills (25, 26); in addition, the literature describes comparisons of self-report questionnaires or clinical interviews by mental health specialists versus clinical evaluation by physicians to determine, specifically, the discriminatory capacity of the latter (25–27). In all these studies, the main conclusions are that there is significant room for improvement in the clinical skills of PHC clinicians.

Globally, OSCEs have been used both to evaluate and to train these skills in a variety of contexts. For example, in the case of nurses who work in outpatient cancer treatment centers, it has been utilized to observe their capacity to recognize and discuss depressive symptoms with patients (28). In addition to this, the studies by Falluco et al. (29, 30), are the only precedents where PHC clinicians have been trained with an OSCE to improve their competence in risk assessment for adolescent depression and suicide. In this case, a training program was designed for these purposes, one of its components being the training of skills with standardized patients through an OSCE (29, 30). This intervention improved the participants’ confidence, knowledge, and clinical practice in relation to risk assessment for adolescent depression and suicide in PHC (29, 30).

These studies have innovated in the evaluation of clinicians’ depression management competences, extending previous uses of OSCEs in educational contexts. In the case of internal medicine residents, this tool has been used to compare how depressive symptoms can affect non-verbal communication and anamnesis in patients with physical comorbidity (31). In addition, OSCEs have been used in mental health training/education programs for nursing students and pediatrics residents, where their potential to improve clinical skills linked to depression management has been demonstrated (32).

Given that the use of OSCEs in the assessment of clinical skills related to depression management in PHC is relatively recent, there is a long road ahead for research and innovation in this area.

### Implications for Practice and Research

Even though depression can be treated effectively by non-specialized health care personnel (33), the detection and management of depression in PHC can be challenging and complex, seriously hindering timely access to treatment (34, 35). In this context, assessment with OSCEs makes it possible to identify clinical and communicative skills of PHC clinicians with a significant margin for improvement, which can be then added to the future curricula of training programs aimed at these professionals.

In this regard, this study revealed that biomedical clinicians performed more poorly in areas such as screening and depression diagnosis. Notably, physicians and nurses not specialized in mental health share a large part of the direct intervention and coordination involved in depression management in PHC, however, the mental health training that these professionals receive amounts to less than 10% of their working hours (19). An education program focused on professional practice that combines additional hours of training in mental health with incentives to improve clinical practice, utilizing OSCEs as a formative tool (29, 30), can significantly improve clinical outcomes.

Another aspect that required additional training was the comprehensive diagnosis of depression and its differential diagnosis in the presence of medical and/or psychiatric comorbidity. In this regard, the presentation of depression in conjunction with physical and/or psychiatric conditions represents a significant challenge for depression detection, diagnosis, and treatment by PHC physicians, who usually spend their consultation time exploring physical symptoms that mask emotional problems (34). Regrettably, comorbidity between depression and other conditions is the norm rather than the exception in PHC, and major efforts will be required for educational and health institutions, as decision makers, to integrate the complex management of depression to education curricula, continuing education, and clinical practice guidelines (36).

Additionally, in this study, the OSCE was able to reveal problems in clinical record keeping. This can negatively impact the continuity of care of patients with depression, becoming an obstacle to better interaction and formal communication between the members of the PHC team, which reduces the quality of teamwork. This has been identified in the literature as a significant barrier to structured and multi-professional management of depression in PHC (37).

Future studies should include bigger and more balanced samples of PHC clinicians, be able to determine how clinical and communicative skills for the management of depression vary by sex, age, hours of mental health training received in undergraduate or continuing health education, years of practice, and type of clinician, among other variables. There is evidence that skills for depression management may depend on certain socio-demographic factors (25) and on a person’s attitude towards mental health topics (38). More extensive knowledge in this area will make it possible to personalize mental health training by adapting currently available educational opportunities to the needs of non-specialized healthcare personnel.

Considering the financial and logistical resources demanded by OSCEs, there may be attractive alternatives for their implementation in PHC, according to the emerging evidence. For instance, carefully trained, incognito, standardized patients making unannounced visits to PHC may avoid the interruptions in usual clinical practice that OSCEs require, thus facilitating the examination of actual practice habits (39). In parallel to this, current technology may enable the automatization, standardization, and mass implementation of evaluation processes through the use of tools such as virtual standardized patients or avatars, which have been recently introduced to medical education (32).

Finally, and given that limited consultation time in PHC could impose restrictions on information collection and registration, it is important for future clinicians’ aids to focus on patient evaluation, integrating technologies to make it easier for them to record, store, and access clinical information in these contexts (40), thus freeing them from some of their significant administrative load.

## Conclusions

The OSCE provided an opportunity to assess in depth the clinical and communicative skills of PHC clinicians for the management of depression. Although the clinicians had an acceptable level of competences, there are still areas that require further attention, such as the screening and diagnosis procedures used by biomedical clinicians. Given the significant involvement of these types of clinicians in the management of depression, better opportunities for mental health training should be available at both undergraduate and continuous health education.

## Data Availability

The datasets generated for this study are available on request to the corresponding author.

## Ethics Statement

The participants were those clinicians who were working in multi-professional teams for the management of depression within the selected PHC clinics and gave informed consent before entering the study. The Ethics Committee of the Faculty of Medicine, Universidad de Chile, granted approval for the study under record number 103-2012.

## Author Contributions

PM coordinated the study, analyzed the data, and wrote the draft of the manuscript. GR conceived and designed the study, carried out parts of the data collection, and critically reviewed the manuscript. VM contributed analysis tools and critically reviewed the manuscript. RM analyzed the data and critically reviewed the manuscript. JC assisted in the coordination of the study, carried out parts of the data collection, and critically reviewed the manuscript. And VG participated in the discussions and helped write the manuscript.

## Funding

This study was funded by CONICYT (National Fund for Science and Technology Research) under project FONDECYT no. 1100205, the Millennium Science Initiative of the Ministry of Economy, Development and Tourism, grant “Millennium Nucleus to Improve the Mental Health of Adolescents and Youths, Imhay” and the Fund for Innovation and Competitiveness (FIC) of the Chilean Ministry of Economy, Development, and Tourism, through the Millennium Science Initiative, Grant No. IS130005. These funding agencies did not contribute to the design or implementation of the study or to the data analysis process, nor did they influence the preparation, review, or approval of the final manuscript.

## Conflict of Interest Statement

The authors declare that the research was conducted in the absence of any commercial or financial relationships that could be construed as a potential conflict of interest.

## References

[1] Mitchell AJ Vaze A Rao S Clinical diagnosis of depression in primary care: a meta-analysis. Lancet (2009) 374:609–19. 10.1016/S0140-6736(09)60879-5 19640579

[2] World Health Organization Depression and other common mental disorders: global health estimates. Geneva: World Health Organization (2017).

[3] Dong JY Zhang YH Tong J Qin LQ Depression and risk of stroke: a meta-analysis of prospective studies. Stroke (2012) 43:32–7. 10.1161/STROKEAHA.111.630871 22020036

[4] Lloyd C Roy T Nouwen A Chauhan A Epidemiology of depression in diabetes: international and cross-cultural issues. J Affect Disord (2018) 2018:22–9. 10.1016/S0165-0327(12)70005-8 23062853

[5] Pan A Sun Q Okereke OI Rexrode KM Hu BF Depression and risk of stroke morbidity and mortality: a meta-analysis and systematic review. JAMA (2011) 306:1241–9. 10.1001/jama.2011.1282 PMC3242806 21934057

[6] Blakemore A Dickens C Guthrie E Bower P Kontopantelis E Afzal C Depression and anxiety predict health-related quality of life in chronic obstructive pulmonary disease: systematic review and meta-analysis. Int J Chron Obstruct Pulmon Dis (2014) 9:501–12. 10.2147/COPD.S58136 PMC4035108 24876770

[7] Moussavi S Chatterji S Verdes E Tandon A Patel V Ustun B Depression, chronic diseases, and decrements in health: results from the world health surveys. Lancet (2007) 370:851–8. 10.1016/S0140-6736(07)61415-9 17826170

[8] Gibb SJ Fergusson DM Horwood LJ Burden of psychiatric disorder in young adulthood and life outcomes at age 30. Br J Psychiatr (2010) 197:122–7. 10.1192/bjp.bp.109.076570 20679264

[9] Riihimäki K Vuorilehto M Isometsä E A 5-year prospective study of predictors for functional and work disability among primary care patients with depressive disorders. Eur Psychiatry (2015) 30:51–7. 10.1016/j.eurpsy.2014.02.005 24721280

[10] Cuijpers P Vogelzangs N Twisk J Kleiboer A Li J Penninx BW Comprehensive meta-analysis of excess mortality in depression in the general community versus patients with specific illnesses. Am J Psychiatry (2014) 171:453–62. 10.1176/appi.ajp.2013.13030325 24434956

[11] Linde K Kriston L Rücker G Jamil S Schumann I Meissner K Efficacy and acceptability of pharmacological treatments for depressive disorders in primary care: systematic review and network meta-analysis. Ann Fam Med (2015a) 13:69–79. 10.1370/afm.1687 25583895 PMC4291268

[12] Linde K Sigterman K Kriston L Rücker G Jamil S Meissner K Effectiveness of psychological treatments for depressive disorders in primary care: systematic review and meta-analysis. Ann Fam Med (2015b) 13:56–68. 10.1370/afm.1719 25583894 PMC4291267

[13] Chisholm D Sweeny K Sheehan P Rasmussen B Smit F Cuijpers P Scaling-up treatment of depression and anxiety: a global return on investment analysis. Lancet (2016) 3:415–24. 10.1016/S2215-0366(16)30024-4 27083119

[14] Hardeveld F Spijker J De Graaf R Hendriks SM Licht CM Nolen WA Recurrence of major depressive disorder across different treatment settings: results from the NESDA study. J Affect Disord (2013) 147:225–31. 10.1016/j.jad.2012.11.008 23218899

[15] Wardenaar KJ Conradi HJ de Jonge P Data-driven course trajectories in primary care patients with major depressive disorder. Depress Anxiety (2014) 31:778–86. 10.1002/da.22228 24390862

[16] Saxena S Thornicroft G Knapp M Whiteford H Resources for mental health: scarcity, inequity, and inefficiency. Lancet (2007) 370:878–89. 10.1016/S0140-6736(07)61239-2 17804062

[17] American Psychiatric Association& Academy of Psychosomatic Medicine Dissemination of integrated care within adult primary care settings: the collaborative care model (2016). Retrieved June 20, 2018, from https://www.psychiatry.org/File%20Library/Psychiatrists/Practice/Professional-Topics/Integrated-Care/APA-APM-Dissemination-Integrated-Care-Report.pdf.

[18] Thota AB Sipe TA Byard GJ Zometa CS Hahn RA Knight-Eily LR Collaborative care to improve the management of depressive disorders: a community guide systematic review and meta-analysis. Am J Preven Med (2012) 42:525–38. 10.1016/j.amepre.2012.01.019 22516495

[19] Pan American Health Organization WHO-AIMS. Report on mental health system in Latin America and the Caribbean. Washington, D.C: Pan American Health Organization (2013).

[20] Epstein RM Assessment in medical education. N Engl J Med (2007) 356:387–96. 10.1056/NEJMra054784 17251535

[21] Rojas G Martínez P Vöhringer PA Martínez V Castro-Lara A Fritsch R Comprehensive technology-assisted training and supervision program to enhance depression management in primary care in Santiago, Chile: study protocol for a cluster randomized controlled trial. Trials (2015) 16:311. 10.1186/s13063-015-0845-4 26201546 PMC4512091

[22] Alvarado R Rojas G Minoletti A Alvarado F Domínguez C Depression program in primary health care: the Chilean experience. Int J Ment Health (2012) 41:38–47. 10.2753/IMH0020-7411410103

[23] Barrows HS Simulated (standardized) patients and other human simulations. Chapel Hill: Health Sciences Consortium (1987).

[24] Lake CR How academic psychiatry can better prepare students for their future patients: part I: the failure to recognize depression and risk for suicide in primary care; problem identification, responsibility, and solutions. Behav Med (2010) 34:95–100. 10.3200/BMED.34.3.95-100 18829423

[25] Acuña J Rdz-Navarro K Huepe G Carcamo M Botto A Jimenez J Clinical skills of Chilean general practitioners for the management of depressive disorders. Rev Méd Chile (2016) 144:47–54. 10.4067/S0034-98872016000100007 26998982

[26] Levav I Kohn R Montoya I Palacio C Rozic P Solano I Training Latin American primary care physicians in the WPA module on depression: results of a multicenter trial. Psychol Med (2005) 35:35–45. 10.1017/S0033291704002764 15842027 PMC2723767

[27] Vöhringer PA Jimenez MI Igor MA Fores GA Correa MO Sullivan MC Detecting mood disorder in resource-limited primary care settings: comparison of a self-administered screening tool to general practitioner assessment. J Med Screen (2013) 20:118–24. 10.1177/0969141313503954 24080916

[28] Brown RF Bylund CL Kline N De La Cruz A Solan J Kelvin J Identifying and responding to depression in adult cancer patients: evaluating the efficacy of a pilot communication skills training program for oncology nurses. Cancer Nurs (2009) 32:E1–7. 10.1097/NCC.0b013e31819b5a76 19295421

[29] Fallucco EM Conlon MK Gale G Constantino JN Glowinski AL Use of a standardized patient paradigm to enhance proficiency in risk assessment for adolescent depression and suicide. J Adolesc Health (2012) 51:66–72. 10.1016/j.jadohealth.2011.12.026 22727079

[30] Falluco EM Seago RD Cuffe SP Kraemer DF Wysocki T Primary care provider training in screening, assessment, and treatment of adolescent depression. Acad Pediatr (2015) 15:326–32. 10.1016/j.acap.2014.12.004 25824896

[31] Crapanzano K Fisher D Hammarlund R Hsieh EP May W Internal medicine residents’ attitudes toward simulated depressed cardiac patients during an objective structured clinical examination: a randomized study. J Gen Intern Med (2018) 33:886–91. 10.1007/s11606-017-4276-7 PMC5975134 29340941

[32] Williams B Reddy P Marshall S Beovich B McKarney L Simulation and mental health outcomes: a scoping review. Adv Simul (2017) 2:2. 10.1186/s41077-016-0035-9 PMC5806484 29450003

[33] Patel V Talking sensibly about depression. PLoS Med (2017) 14:e1002257. 10.1371/journal.pmed.1002257 28376089 PMC5380305

[34] Barley EA Murray J Walters P Tylee A Managing depression in primary care: a meta-synthesis of qualitative and quantitative research from the UK to identify barriers and facilitators. BMC Fam Pract (2011) 12:47. 10.1186/1471-2296-12-47 21658214 PMC3135545

[35] O’Connor EA Whitlock EP Beil TL Gaynes BN Screening for depression in adult patients in primary care settings: a systematic evidence review. Ann Intern Med (2009) 151:793–803. 10.7326/0003-4819-151-11-200912010-00007 19949145

[36] Martínez P Rojas G Fritsch R Martínez V Vöhringer P Castro A Comorbidity in people with depression seeking help at primary health care centers in Santiago, Chile. Rev Méd Chile (2017) 145:25–32. 10.4067/S0034-98872017000100004 28393966

[37] Wood E Ohlsen S Ricketts T What are the barriers and facilitators to implementing collaborative care for depression? A systematic review. J Affect Disord (2017) 214:26–43. 10.1016/j.jad.2017.02.028 28266319

[38] Shirazi M Lonka K Parikh SV Ristner G Alaeddini F Sadeghi M A tailored educational intervention improves doctor’s performance in managing depression: a randomized controlled trial. J Eval Clin Pract (2013) 19:16–24. 10.1111/j.1365-2753.2011.01761.x 21883718

[39] Shirazi M Sadeghi M Emami A Kashani AS Parikh S Alaeddini F Training and validation of standardized patients for unannounced assessment of physicians’ management of depression. Acad Psychiatry (2009) 35:382–7. 10.1176/appi.ap.35.6.382 22193736

[40] Hollis C Morriss R Martin J Amani S Cotton R Denis M Technological innovations in mental healthcare: harnessing the digital revolution. Br J Psychiatr (2015) 206:263–5. 10.1192/bjp.bp.113.142612 25833865

